# Fusion of Chitin-Binding Domain From *Chitinolyticbacter meiyuanensis* SYBC-H1 to the Leaf-Branch Compost Cutinase for Enhanced PET Hydrolysis

**DOI:** 10.3389/fbioe.2021.762854

**Published:** 2021-12-15

**Authors:** Rui Xue, Yinping Chen, Huan Rong, Ren Wei, Zhongli Cui, Jie Zhou, Weiliang Dong, Min Jiang

**Affiliations:** ^1^ State Key Laboratory of Materials-Oriented Chemical Engineering, College of Biotechnology and Pharmaceutical Engineering, Nanjing Tech University, Nanjing, China; ^2^ Junior Research Group Plastic Biodegradation, Department of Biotechnology and Enzyme Catalysis, Institute of Biochemistry, University of Greifswald, Greifswald, Germany; ^3^ Key Laboratory of Agricultural Environmental Microbiology, College of Life Science, Nanjing Agriculture University, Nanjing, China

**Keywords:** chitin-binding domain, polyethylene terephthalate, hydrolysis, leaf-branch compost, hydrophobicity

## Abstract

Polyethylene terephthalate (PET) is a mass-produced petroleum-based non-biodegradable plastic that contributes to the global plastic pollution. Recently, biocatalytic degradation has emerged as a viable recycling approach for PET waste, especially with thermophilic polyester hydrolases such as a cutinase (LCC) isolated from a leaf-branch compost metagenome and its variants. To improve the enzymatic PET hydrolysis performance, we fused a chitin-binding domain (ChBD) from *Chitinolyticbacter meiyuanensis* SYBC-H1 to the C-terminus of the previously reported LCC^ICCG^ variant, demonstrating higher adsorption to PET substrates and, as a result, improved degradation performance by up to 19.6% compared to with its precursor enzyme without the binding module. For compare hydrolysis with different binding module, the catalytic activity of LCC^ICCG^-ChBD, LCC^ICCG^-CBM, LCC^ICCG^-PBM and LCC^ICCG^-HFB4 were further investigated with PET substrates of various crystallinity and it showed measurable activity on high crystalline PET with 40% crystallinity. These results indicated that fusing a polymer-binding module to LCC^ICCG^ is a promising method stimulating the enzymatic hydrolysis of PET.

## Introduction

Plastics are being used in an increasing number of applications in our society. As a result, improperly disposed waste plastics have resulted in environmental pollution, which has garnered increasing attention in recent decades. According to recent data, global plastics production has reached nearly 368 million tons in 2019 (PlasticsEurope, 2020). However, 70% of plastic waste is landfilled or discarded carelessly, 11% is incinerated, and only 19% is recycled (Kaza et al., 2018). Polyethylene terephthalate (PET) is a thermoplastic polyester synthesized with the monomers terephthalic acid (TPA) and ethylene glycol (EG). PET has been widely used in the production of beverage bottles and synthetic fibers because of its excellent mechanical and thermal properties (Wei and Zimmermann, 2017; Kawai et al., 2019; Chen et al., 2020). Chemical and mechanical recycling of PET involve harsh chemicals and energy-intensive physicochemical treatments (Ragaert et al., 2017). Enzymatic hydrolysis under mild reaction conditions has recently been recognized as an eco-friendly alternative recycling process for PET (Wei et al., 2020). PET-hydrolyzing enzymes, which belong to different subclasses of the alpha-beta hydrolase family, are the most extensively studied plastic-degrading enzymes (Chen et al., 2020). The first reported PET hydrolase was TfH from *Thermobifida fusca* which can degrade pretreated PET waste at 55 °C (Müller et al., 2005). A synergy between different hydrolase subclasses, for example a polyester degrading *Is*PETase and an oligomer degrading *Is*MEHTase, has been reported in the bacterium *Ideonella sakaiensis*, which is capable of metabolizing amorphous PET (Yoshida et al., 2016). Tournier et al. recently demonstrated the use of an engineered PET hydrolase variant in the degradation of pre-treated post-consumer PET bottles, resulting in a >90% depolymerization of PET waste in less than 10 h at an industrially relevant scale. This equates to a mean productivity of 16.7 g L^−1^ h^−1^ of TPA which was then recovered to synthesize virgin polymers, thereby closing the recycling loop (Tournier et al., 2020). This ground-breaking innovation developed by the French biotech company Carbios used the highly efficient wild-type the leaf-branch compost cutinase (LCC) (Sulaiman et al., 2012) for protein engineering, and thermomechanical pretreatment to reduce the crystallinity of the real-world PET waste. This procedure successfully showcased a closed-loop bio-recycling PET that holds great promise as a foundation for future applications (Wei et al., 2020).

As a surface erosion process, enzymatic PET hydrolysis can be significantly accelerated when more biocatalysts can adsorb to the hydrophobic polymer surface (Kawai et al., 2019). Hydrophobic binding modules have been fused to selective PET hydrolyzing enzymes in order to improve the enzyme sorption at water-solid interface and thus facilitate the hydrolysis of insoluble polymer substrates (Din et al., 1991; Ribitsch et al., 2013). Ribitsch et al. (2015) engineered the PET cutinase Thc_Cut1 by fusing it with two *Trichoderma hydrophobins* HFB4 and HFB7, resulting in a more than 16-fold increase in PET hydrolysis efficiency with the fusion variants. Similarly, they were able to modify the same enzyme by fusing two other binding domains: the carbohydrate binding modules (CBM) from *Hypocrea jecorina* and the polyhydroxyalkanoate binding modules (PBM) from *Alcaligenes faecalis*, resutling in an enhanced hydrolysis activity on amorphous PET of up to 3.8 times (Ribitsch et al., 2013).

Plastic polymers and plants (such as cellulose and chitin) share chemical properties and polymer structures, including partially crystalline regions, surface hydrophobicity and backbones linked by hydrolysable bonds (Daly et al., 2021). The carbohydrate binding modules (CBM), which includes the chitin-binding domain (ChBD), is a type of noncatalytic domain found extensively in glycoside hydrolases facilitating their adsorption to the insoluble substrate (Boraston et al., 2004). CBMs have been developed as useful affinity tags for protein immobilization due to their high adsorbing capacity to solid materials. For example, Zhou et al. (2020) used chitin-binding domain as an affinity tag and achieved over 95% immobilization efficiency with their target protein. Furthermore, CBMs have a broad range of hydrophobic binding affinity with many polymer substrates including cellulose, chitin, xylan, and starch (Oliveira et al., 2015).

**FIGURE 1:**
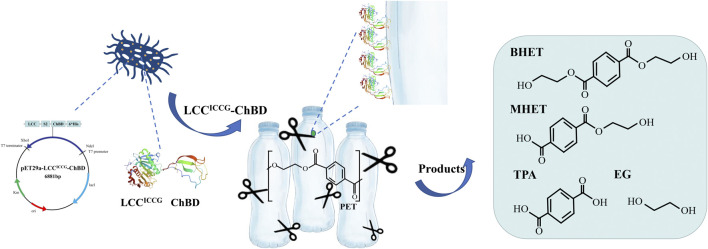
Schematic illustration of the degradation of PET using the recombinant LCC^ICCG^-ChBD expressed in *E. coli*.

In this study, protein mutants were constructed based on the PET hydrolase variant LCC^ICCG^, which has been shown by Tournier et al. as the most active PET hydrolase to date, with C-terminally fused binding modules including a ChBD from *Chitinolyticbacter meiyuanensis* SYBC-H1 (Zhang et al., 2018), the carbohydrate binding modules (CBM) from *Hypocrea jecorina*, the polyhydroxyalkanoate binding modules (PBM) from *Alcaligenes faecalis* and *Trichoderma* hydrophobins HFB4. While the latter three binding modules have been used to engineer other PET hydrolases, the ChBD was introduced to PET hydrolases for the first time due to its similar hydrophobic nature to CBM, which is mediated by tryptophan residues and can promote the enzyme adsorption and, as a result, the hydrolytic activity on PET (Ribitsch et al., 2013). To investigate the effect of the binding module on the PET-hydrolyzing cutinase, the degradation efficiency with LCC^ICCG^ and its fusion variants on various PET materials were evaluated in terms of the surface adsorption, release of the degradation products and kinetic analysis (Figure 1).

## Materials and Methods

### Reagents and Strains

Amorphous PET film (GF-PET, crystalline 6.7%) was purchased from Goodfellow Ltd. (Shanhai, China, thickness of 250 µm). Post-consumer PET waste (PCW-PET, crystalline 16%) samples were prepared from PET flakes following the pretreatment method described previously (Tournier et al., 2020). High-crystalline PET (Hc-PET, crystalline 40%) was purchased from Dopont Ltd. (Shanghai, China). All PET samples were cut into pieces then immersed in liquid nitrogen for 1 min before micronization. The percentage crystallinity of the sample is measured by the DSC method and calculated (Tournier et al., 2020). MHET is synthesized based a previously described method (Kaabel et al., 2021). All other chemicals and reagents were of analytical grade and purchased from Sahn chemical technology Co. Ltd. (Shanghai, China). Restriction enzymes, DNA polymerase were purchased from TaKaRa Co., Ltd. (Dalian, China). *Escherichia coli* strains were routinely cultivated aerobically in LB medium at 37°C. All constructs in this study were generated with the expression vector pET-29a for recombinant expression in *E. coli* BL21 (DE3). The gene encoding LCC^ICCG^ cutinase, CBM, PBM and HFB4 were synthesized based on previous studies (Ribitsch et al., 2013; Ribitsch et al., 2015; Tournier et al., 2020) and that for ChBD was derived from the genome of *Chitinolyticbacter meiyuanensis* SYBCH1 (Zhang et al., 2018). LCC^ICCG^ cutinase and ChBD expression strain *E. coli* BL21 (pET29a-LCC^ICCG^) and *E. coli* BL21 (pET29a-ChBD) were constructed in previous experiments (Zhou et al., 2020).

### Construction of Chimeric Genes and Recombinant Plasmids

**FIGURE 2:**
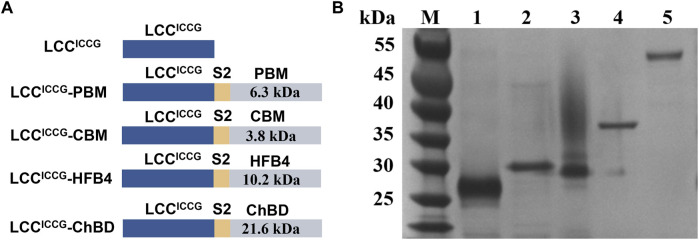
**(A)** Schematic illustration of the constructed fusion enzymes based on LCC^ICCG^ with ChBD; **(B)** SDS-PAGE analysis (12% polyacrylamide gel) of the purified fusion proteins expressed in *E. coli* BL21 (DE3): 1. LCC^ICCG^ 2. LCC^ICCG^-PBM 3. LCC^ICCG^-CBM 4. LCC^ICCG^-HFB4 5. LCC^ICCG^-ChBD.

The genes were constructed using the method of splicing-by-overlap extension (Su et al., 2006). Synthetic oligonucleotides encoding sequences of peptide linkers was introduced between the *lcc* and binding module genes. To express the fusion enzymes, the cleavage sites for the restriction enzymes *Nde I* and *Xho I* were introduce into the 5′ end of *lcc* and the 3′end of binding module genes, respectively (Figure 2A). The gene fragments encoding GFP was then inserted into the multi clone site (MCS) by one step cloning for further investigations.

### Enzyme Expression and Purification

*E. coli* BL21 (DE3) harboring individual plasmids including pET29a-LCC^ICCG^, pET29a-LCC^ICCG^-ChBD, pET29a-LCC^ICCG^-CBM, pET29a-LCC^ICCG^-PBM, pET29a-LCC^ICCG^-HFB4 and pET29a-LCC^ICCG^-ChBD-GFP was used to inoculate 5 ml LB medium containing kanamycin (50 μg/ml) for incubation at 37°C and 200 rpm for 12 h. Then, a 2 ml seed culture solution was removed to inoculate 200 ml LB medium with kanamycin (50 μg/ml) for incubation at 37°C and 200 rpm for 4 h. When the OD value reached 0.6, IPTG was added at a final concentration of 1 mM to induce the recombinant protein expression by further incubating at 18°C for 24 h. The bacterial cells were harvested by centrifugation at 12,000 rpm at 4°C for 10 min. The obtained cell pellets were washed twice by phosphoric buffer (50 mM, pH 8.0) and resuspended with 50 mM phosphoric buffer (pH 8.0). Then, the cells were disrupted by a scientz-II D ultrasonic generator and cell debris was removed by centrifugation at 12,000 rpm and 4°C for 20 min. The recombinant enzyme with a C-terminal His6-tag was purified using Ni^2+^-NTA resin with a Biomolecular Liquid Chromatography System (AKTA design, GE Healthcare, US). The target proteins were eluted with buffer NPI-250 (50 mM NaH_2_PO_4_, 300 mM NaCl, 250 mM imidazole, pH 8.0). The purified protein was collected and concentrated by ultrafiltration tube to remove imidazole. Esterase activity was determined using a slightly modified version of a previously described method (Alisch et al., 2004). In a cuvette, 980 μL of 50 mM PBS buffer (K_2_HPO_4_/NaH_2_PO_4_, pH 8.0) was mixed with 10 μL *p*-nitrophenyl butyrate (*p*NPB, 10 mM) dissolved in isopropanol. The hydrolysis reaction was started by adding 10 μL of appropriately diluted enzyme solution and stopped by adding 20 μL of 1% sodium dodecyl sulfonate, after which the absorbance was measured at 410 nm.

### Kinetic Studies

The kinetic parameters for the enzymatic hydrolysis of *p*NPB were calculated based on the reaction rates determined in the initial reaction phase during first 3 min reaction. The kinetic parameters for hydrolysis of amorphous PET film which was cut into pieces then immersed in liquid nitrogen for 1 min and micronization. obtained in the initial phase during a reaction time of 30 min was used to determine the reaction rates. Further, this amount was subtracted from the sum of the total soluble hydrolysis products (TPA, MHET and BHET) obtained to calculate the reaction rates.

### Enzyme-PET Binding Assay

The purified enzyme (LCC^ICCG^-ChBD-GFP) and the pre-treated PET substrate were mixed thoroughly for 10 min, and centrifuged at 12,000 rpm for 10 min at 4°C. The adsorption efficiency of the binding module to the plastic substrate was calculated based on the protein concentrations determined in the supernatant before and after mixing and incubation. Experiments on this adsorption efficiency were carried out at different incubation temperatures (4, 20, 40, and 65°C) to monitor the maximum binding efficiency of different enzyme variants at the temperature where the plastic degradation was performed.

Protein concentrations were determined by the Bradford method (Campion et al., 2017). The amount of adsorbed ChBD-GFP was determined using the fluorescence intensity detected by Hitachi F7000 fluorescence spectrophotometer similarly as describe before (Cha and Kwon, 2018). The adsorption rate was calculated with the formula below:

$$\mathrm{Adsorption}\;\mathrm{rate}=\left(1-\frac{\mathrm{GFP}\;\mathrm{concentration}\;\mathrm{in}\;\mathrm{supernatant}}{\mathrm{Initial}\;\mathrm{GFP}\;\mathrm{concentraion}}\right)\times \mathrm{100}\%$$

### Hydrolysis of the PET Substrates

The enzymatic degradation of PET materials was carried out in a 500 ml reactor. The reaction began with the addition of 0.5 μmol of enzyme to 200 ml buffer (100 mM potassium phosphate buffer pH 8) containing 120 mg of PET substrate, which was then incubated at 65°C under stirring at 100 rpm for 12 h. The enzymatic depolymerization efficiency was calculated using the sum amount of BHET, MHET and TPA released into the reaction supernatants as measured by HPLC. For this calculation, the average molecular weight of one repeating unit of the PET polymer of 192.2 g/mol was used (Yan et al., 2021).

### HPLC Analysis

The samples were filtered through 0.22 μm filters and analyzed by a Shimadzu CMB-20 A HPLC system coupled with a C18 column (InerSustain, 4.6 × 250 mm, 5 μm). The C18 column was eluted with solvent (18% acetonitrile, 1% formic acid and 81% water, pH 2.5) in 0–25 min. The samples were monitored at 254 nm.

## Result

### Cloning, Expression, and Purification of the Fusion Proteins

The gene encoding LCC^ICCG^-ChBD, LCC^ICCG^-CBM, LCC^ICCG^-PBM and LCC^ICCG^-HFB4 were cloned into the expression vector pET29a. The peptide S2 was used as a linker between the PET hydrolase and the binding module. An additional construct pET29a-LCC^ICCG^-ChBD-GFP with a gene encoding the green fluorescent protein (GFP) at a 3’ downstream position to ChBD was also created for fluorescent detection of the recombinant proteins (Figure 2A). SDS-PAGE in Figure 2B revealed that the LCC^ICCG^ variant, as well as the fusion proteins were obtained as highly purified recombinant, respectively.

### Kinetic Analyses of the Hydrolysis of *p*NPB and Amorphous PET Film

The kinetic parameters for the enzymatic hydrolysis of *p*NPB were determined for the fusion proteins and compared to those obtained with LCC^ICCG^ (Table 1). The *K*_*m*_ values obtained with the fusion proteins were significantly higher, while the corresponding *k*_*cat*_ */K*_*m*_ value was approximatively 20% less than that of LCC^ICCG^. But instead, the kinetic parameters with amorphous PET film as substrate shows that the fusing proteins LCC^ICCG^-ChBD have low *K*_*m*_ values (131.9 μM) compared to LCC^ICCG^ (171.8 μM) mearing better substrate affinity to amorphous PET film. At the same time, LCC^ICCG^-ChBD have about 2 times *k*_*cat*_ */K*_*m*_ than the cutinase without binding module (Table 2).

**TABLE 1:** Kinetic parameters for the hydrolysis of *p*NPB catalyzed by LCC^ICCG^ and LCC^ICCG^-ChBD.

Enzyme	_*Km* (μM)_	*k*_*cat*_ (s^−1^)	*k*_*cat*_/*K*_*m*_ (s^−1^/μM)
LCC^ICCG^	126.3	67.5	0.534
LCC^ICCG^-ChBD	141.8	68.4	0.404

**TABLE 2:** Michalies-Menten kinetic parameters for the hydrolysis of amorphous PET catalyzed by LCC^ICCG^ and LCC^ICCG^-ChBD.

Enzyme	_*Km* (μM)_	*k*_*cat*_ (s^−1^)	*k*_*cat*_/*K*_*m*_ (s^−1^/μM)
LCC^ICCG^	171.8	30.6	0.178
LCC^ICCG^-ChBD	131.9	45.2	0.343

### Adsorption Rates Monitored by Fluorescence Detection

**FIGURE 3:**
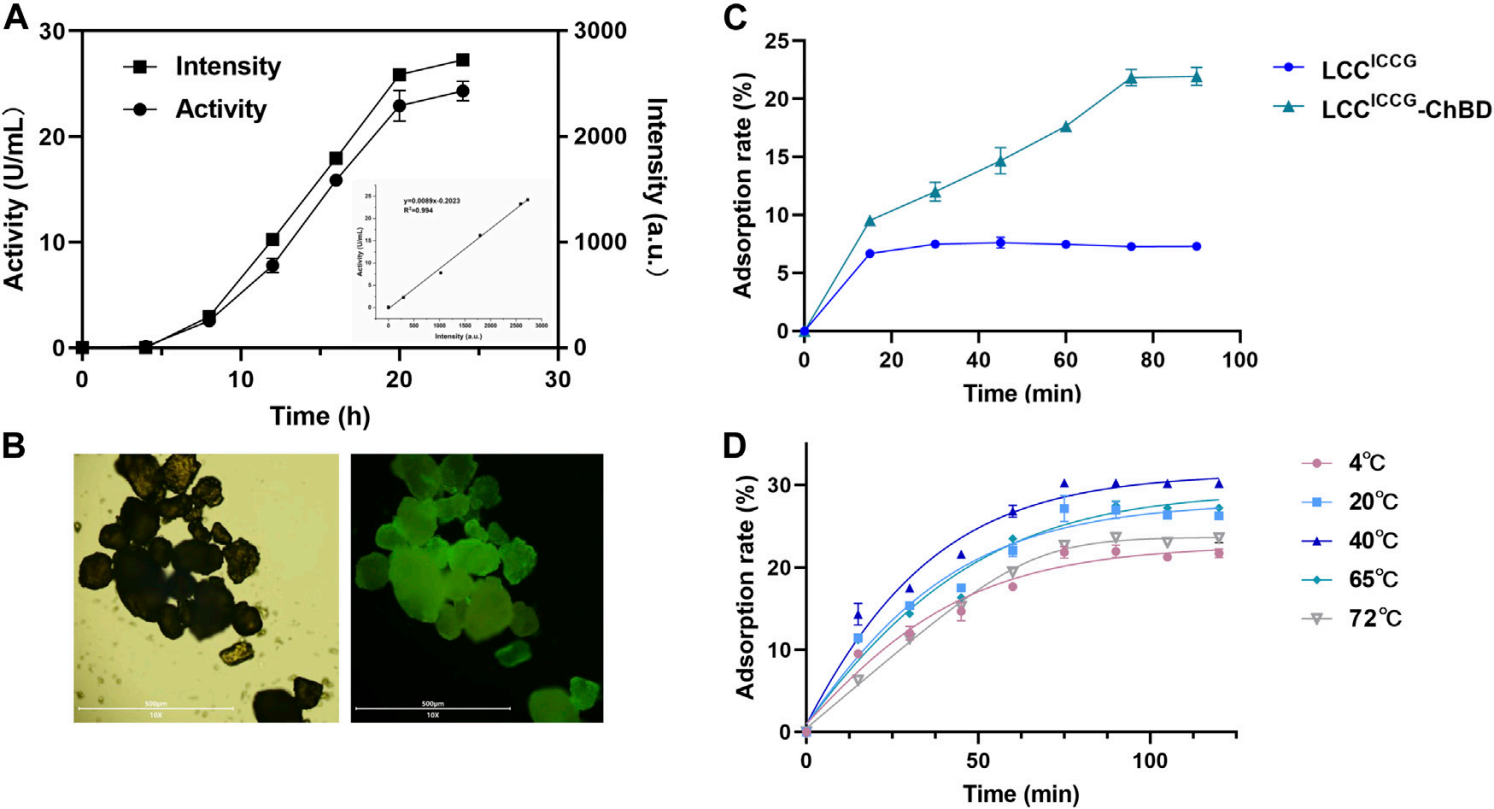
Time courses of production **(A)** and the adsorption of LCC^ICCG^ and LCC^ICCG^-ChBD to GF-PET films monitored by fluorescence analysis **(B,C)** and at different incubation temperatures **(D)**.

Figure 3 showed the time courses of adsorption rates of PET hydrolases with and without ChBD determined fluorimetrically (Figure 3B). The GFP fluorescence and LCC activity showed a decent linear relationship within 24 h (Figure 3A). After 80 min of incubation at 4°C, the maximum adsorption rate of 22.3% was obtained with LCC^ICCG^-ChBD. By contrast, the saturated adsorption rate with LCC^ICCG^ was approximately 7% which has been achieved already after 30 min of incubation (Figure 3C). As shown in Figure 3D, the adsorption time courses with LCC^ICCG^-ChBD were investigated at various temperatures. At 40°C, the maximum adsorption efficiency of 30.2% was achieved after 70 min of incubation. This is the optimal binding temperature for the chitin binding domain used in this study according to previous publications (Zhou et al., 2020). With increasing incubation temperature to 65°C or 72°C, the maximum adsorption rate decreased slightly to 27% or 23.5%, which was achieved after 2 h incubation and considered beneficial for the enzymatic plastic degradation carried out at this temperature.

### Degradation Time Course of GF-PET Powder by Fusion Enzymes

**FIGURE 4:**
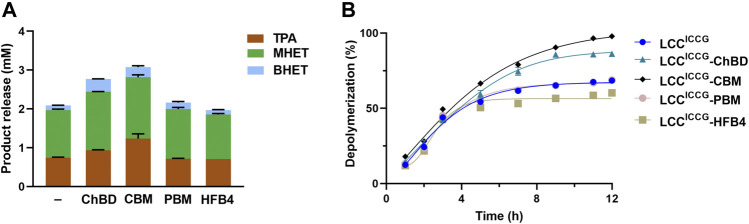
Time courses of hydrolysis product release from ground GF-PET powder by fusion enzymes determined at 65°C and pH 8.

Figure 4 showed the time course study of the GF PET degradation by the cutinase variant with and without various binding modules in terms of the release of HPLC detectable hydrolysis products. In all samples, mono (2-hydroxyethyl) terephthalate (MHET) is the most abundant product followed by terephthalic acid (TPA), and bis (2-hydroxyethyl) terephthalate (BHET). During the total incubation time up to 12 h, the levels of released TPA and MHET increased continuously. After 12 h reaction at 65°C, LCC^ICCG^ yielded 2.12 mM hydrolysis products composed of 0.76 mM TPA, 1.25 mM MHET and 0.11 mM BHET (Figure 4A). LCC^ICCG^-ChBD showed a higher activity against the GF-PET, releasing 0.92 mM TPA, 1.5 mM MHET and 0.32 mM BHET after 12 h incubation, accounting for 29% more yield in total product amount than that without binding module (Figure 4A). In comparison, the highest amount of degradation products composed of 1.23 mM TPA, 1.56 mM MHET and 0.25 mM BHET was yielded with LCC^ICCG^-CBM, and is 14% higher than with LCC^ICCG^-ChBD (Figure 4A). The yield of degradation products with LCC^ICCG^-PBM and LCC^ICCG^-HFB4 was comparable or slightly lower than that with LCC^ICCG^, indicating that the fusion of PBM and HFB4 to LCC^ICCG^ might have weakly impaired its catalytic activity. As shown in Figure 4B, depolymerization degrees of 87.5 and 98.5% were achieved with LCC^ICCG^-ChBD and LCC^ICCG^-CBM, respectively, which are 19.6 and 30.6% higher than that obtained with the enzyme without binding modules (Figure 4B).

### Hydrolysis of PET Substrates With Different Crystallinity

**FIGURE 5:**
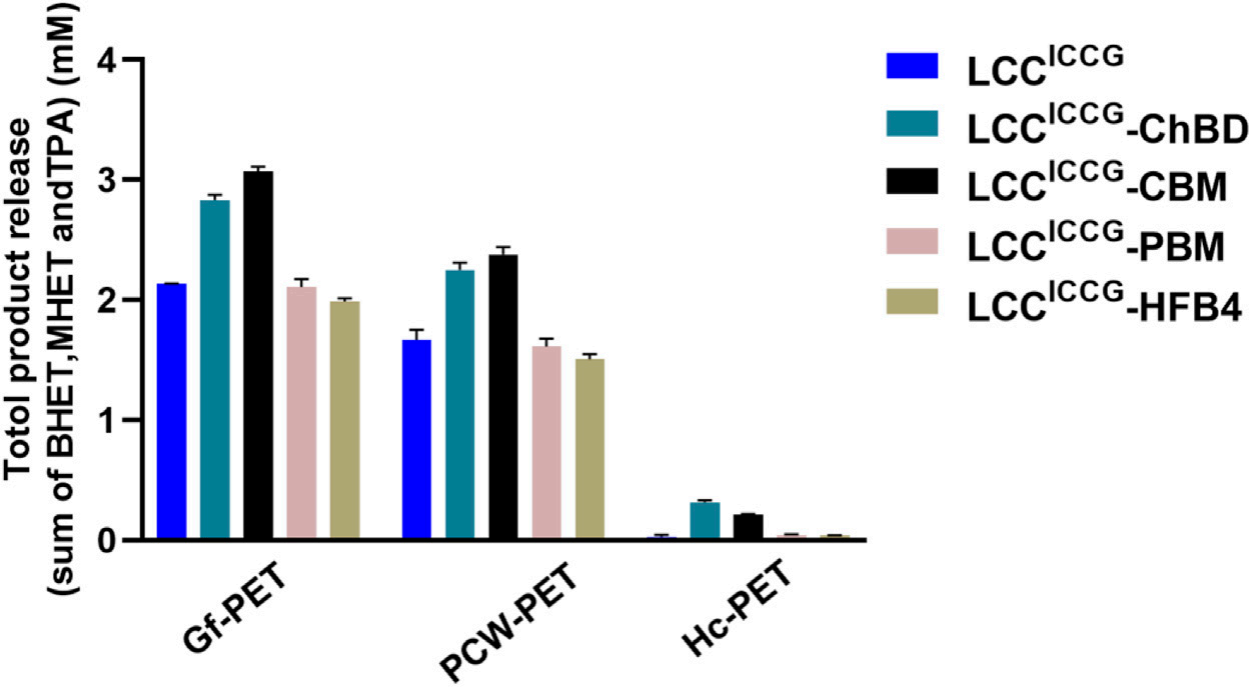
Release of degradation products from PET samples with various crystallinity exposed to fusion enzymes as a function of incubation time as well as converted to the rate of depolymerization.

PET substrates with different crystallinity, such as GF-PET (6.7%), Pcw-PET (16%) and Hc-PET (40%), were used to investigate the effect of crystallinity on the degradation performance catalyzed by various cutinase versions. Figure 5 indicated that the effectiveness of enzymatic hydrolysis decreased significantly as polymer crystallinity increased. LCC^ICCG^, in particular, was nearly impossible to break down Hc-PET exhibiting only 0.029 mM hydrolysis products released after 12 h. Interestingly, the fusion enzymes LCC^ICCG^-ChBD and LCC^ICCG^-CBM showed markedly increased depolymerization performance against Hc-PET by releasing 0.335 and 0.215 mM hydrolysis products, respectively, which are 11.6 and 7.1 times higher than without binding modules. The superior activity of LCC^ICCG^-ChBD and LCC^ICCG^-CBM was noticeable against all three PET substrates with different crystallinity. Furthermore, ChBD outperform other binding modules in facilitating the breakdown of Hc-PET materials.

### Scanning Electron Microscopy Analysis of GF-PET Film Exposed to LCC^ICCG^-ChBD

**FIGURE 6:**
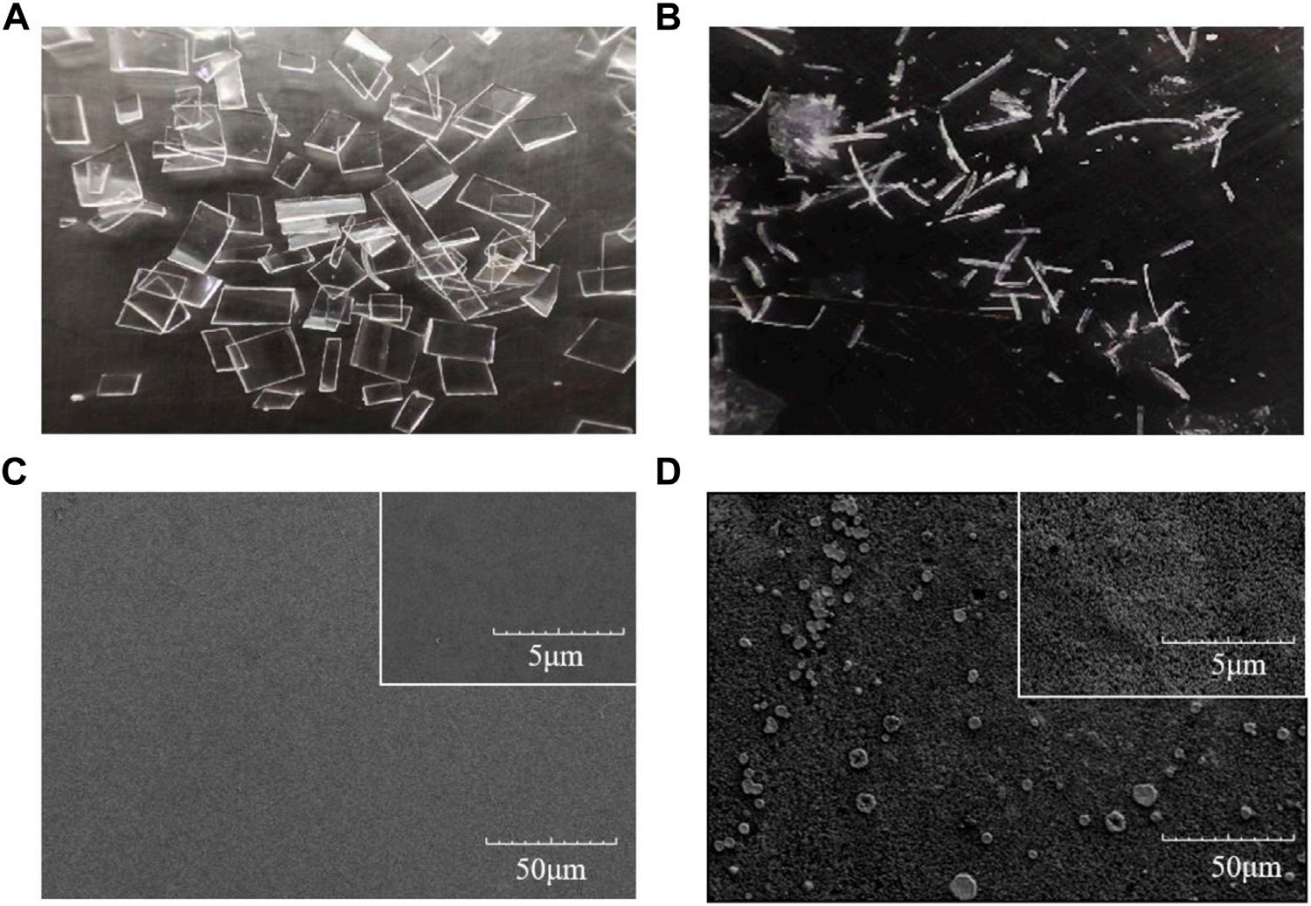
Images of GF-PET films captured by a conventional camera **(A,B)** and by SEM **(C,D)** before **(A–C)** and after enzymatic hydrolysis **(B–D)** with LCC^ICCG^-ChBD.

The degradation of GF-PET film with LCC^ICCG^-ChBD was carried out at 65 °C for 6 h. As shown in Figure 6A, GF-PET is cut into 2*2 cm pieces, and Figure 6B shows the residual plastic fragments after enzymatic breakdown. Figure 6C shows a SEM image of a plastic sheet prior to the degradation, with the surface appearing smooth. A SEM image of a GF-PET film after degradation with increased surface roughness is shown in Figure 6D.

## Discussion

Carbohydrate-binding module (CBM) including ChBD have a high adsorption capacity to a variety of insoluble substrates. These characteristics enable the potential application CBM proteins function as adhesive in food industry, biomedicine, environmental protection and molecular biology. In this study, we employed ChBD to construct a novel fusion enzyme variant for enhanced hydrolysis of synthetic polyesters. To that purposed, an engineered cutinase (LCC^ICCG^) (Tournier et al., 2020) previously published for PET hydrolysis was fused with a C-terminal chitin-binding module from chitinase CmChi1 from *Chitinolyticbacter meiyuanensis* SYBCH1 (LCC^ICCG^-ChBD). This ChBD belongs to carbohydrate-binding modules, which have a similar interaction mechanism with insoluble polymers that involves hydrophobic interactions through tryptophan residues. The ChBD is fused to LCC^ICCG^ *via* a linker region (S2), which has been described as an appropriate linker peptide that can be introduced between distinct enzymes to avoid folding interference from each other allowing the two moieties of a fusion enzyme to function as independently as possible (Lu and Feng, 2008).

The hydrolysis of GF-PET, an amorphous PET material commonly used to study other PET hydrolases, showed intriguing mechanistic insights based on varied ratios of hydrolysis products released by the fusion enzyme and its precursor (Wei et al., 2019; Tiso et al., 2021; Yan et al., 2021). When compared to LCC^ICCG^-ChBD, the one without binding module produced lower amounts of hydrolysis products. LCC^ICCG^ initially released comparable amounts of all hydrolysis products, but the levels of increasing release of MHET and TPA were comparable later in the incubation stage. The ratios of MHET and TPA were nearly unchanged following hydrolysis, regardless of whether the fused protein or its precursor was used. This suggests that the inclusion of the chitin binding module may aid in the depolymerization of the oligomeric PET-related molecules. The results of kinetic characterization further support this view. LCC^ICCG^-ChBD showed a lower *K*_*m*_ value for the *p*NPB hydrolysis than that with its precursor enzyme, indicating a reduced substrate affinity to the soluble model compound. As a result, the catalytic efficiency (*k*_*cat*_ */K*_*m*_) of the enzyme carrying ChBD (0.404 s^−1^/μM) was slightly lower than that measured for LCC^ICCG^ (0.534 s^−1^/μM). LCC^ICCG^-ChBD, on the other hand, has a higher catalytic efficiency (*k*_*cat*_ */K*_*m*_) and depolymerization rate on polymers with various crystallinity than the one without binding module. In general, the addition of ChBD did not appear to considerably improve the depolymerization of PET-related oligomers, although the enhanced depolymerization efficiency of PET polymers was more pronounced. This could be related to the mechanism of the binding module which can potentially destroy the structures of the polymers like celluloses, releasing more accessible bonds to the enzymatic degradation (Din et al., 1991). However, as for PET polymers, more experimental evidence in this context is still required.

In this study, we also compared the promoting effect of the ChBD *Chitinolyticbacter meiyuanensis* with three other binding domains: cellulose–binding domain (CBM) of cellobiohydrolase I from *Trichoderma reesei* (TrCBH), polyhydroxyalkanoate binding modules (PBM) from *Alcaligenes faecalis* and hydrophobin HFB4 from *T. reesei* QM6a, which have been previously reported to significantly improve PET degradation efficiency when fused to another PET hydrolase. By fusing to the hydrolase variant LCC^ICCG^, our results (Figures 4, 5) showed that CBM and ChBD can enhance the hydrolysis of PET samples with different crystallinity. When high-crystalline PET was used as the substrate, the enzyme with ChBD fusion released 11.6 times more degradation products, indicating its superiority in promoting the breakdown of Hc-PET materials. With amorphisized PET sample, the increased degradation performance by 19.6% with the fused ChBD could be attributed to an increased adsorption capacity to PET by up to 27%. As a result, more research is needed to understand why ChBD improves PET hydrolase to different levels against PET with varying crystallinity. In addition, mutagenesis of ChBD by protein engineering approaches may facilitate to gain a better knowledge on its binding process to PET, similarly as previously reported with other CBMs (Weber et al., 2019). Because ChBD has an optimal temperature for PET binding at 40°C, which is significantly lower than the optimal breakdown temperature of PET catalyzed by LCC variants, enhancing the thermal stability of ChBD may be another useful strategy for improving the overall degradation efficacy catalyzed by the fusion protein.

## Conclusion

We have successfully fused a chitin binding domain to LCC^ICCG^, a cutinase variant with outstanding PET hydrolytic activity. The fusion protein was functionally expressed in *E. coli* and showed an enhanced adsorption capacity to PET films by fluorescence detection. As a result, the fusion protein with ChBD demonstrated improved degradation performance on all investigated PET materials as indicated by the higher release of degradation products than that obtained with LCC^ICCG^. The improved hydrolytic activity of LCC^ICCG^-ChBD led to detectable degradation performance on PET substrates with a high crystallinity of 40%.

## Data Availability

The original contributions presented in the study are included in the article/Supplementary Material, further inquiries can be directed to the corresponding authors.
